# Alterations and Interchange of Morphometric Characters in Different Life Cycle Stages with Reference to Genomic Variations of *Anopheles subpictus* (Diptera; Culicidae) Sibling Species Complex in Sri Lanka

**DOI:** 10.3390/insects9030089

**Published:** 2018-07-24

**Authors:** Dona P. W. Jayatunga, Iresha N. Harischandra, Naduviladath V. Chandrasekharan, Nissanka K. de Silva

**Affiliations:** 1 Center for Biotechnology, Department of Zoology, Faculty of Applied Sciences, University of Sri Jayewardenepura, Nugegoda 10250, Sri Lanka; pamodajayatunga@gmail.com (D.P.W.J.); iresha@sci.sjp.ac.lk (I.N.H.); 2 Department of Chemistry, Faculty of Science, University of Colombo, Colombo 03 00100, Sri Lanka; vishvanathc@hotmail.com

**Keywords:** *Anopheles subpictus*, sibling species, morphological variations, molecular markers, Sri Lanka

## Abstract

The species complex of the mosquito *Anopheles subpictus* is designated by the sibling species A–D, depending on morphological characters of life cycle stages and variations in polytene chromosomes. However, morphological aberrations in the life cycle stages make the identification of sibling species uncertain and imprecise. The objective of the present study is to determine the suitability of morphological variations of sibling species and their genomic variations to identify the sibling species status of an *An. subpictus* population in Sri Lanka. Life cycle stages of larvae, pupal exuviae, and adults were examined for previously reported distinctive morphological features. Five nuclear and mitochondrial genome regions, including the Internal transcribed spacer 2 (ITS2) region, D3 region, *white* gene, cytochrome *c* oxidase I (*COI*), and Cytochrome b (*Cyt-b*), were sequenced and analyzed for variations. The eggs changed their distinct sibling morphological characters during metamorphosis (89.33%). The larvae, pupal exuviae, and adult stages showed deviation from their sibling characters by 26.10%, 19.71%, and 15.87%, respectively. However, all the species from the analysis shared two distinct sequence types for all regions, regardless of the morphological variations. In conclusion, the *An. subpictus* sibling species complex in Sri Lanka is not identifiable using morphological characters due to variations, and the genomic variations are independent from the morphological variations.

## 1. Introduction

The *Anopheles* (*Cellia*) *subpictus sensu lato* (*s.l.*) Grassi 1899 species complex is the most abundant anopheline mosquito in the Indian subcontinent [1,2]. It acts as a vector for malaria and Japanese encephalitis in many parts of Asia [2]. *An. subpictus* is the major secondary vector of malaria in Sri Lanka [1,3].

In India, *An. subpictus* was first deemed to be a species complex based on differences in larval morphology [4]. Further, the occurrence of two distinct types of eggs and cytological evidence has temporarily designated two forms of *An. subpictus* sibling species as A and B in India [5]. This taxon has further been categorized as a complex of four sibling species—designated as A, B, C, and D—based on stage-specific morphometric characters [6]. The presence of four sibling species (A–D) has been confirmed through fixed inversions in the X-arm of polytene chromosomes viz. A = X+^a^,+^b^; species B = Xa,b; species C = Xa,+^b^; species D = X + a, b [6]. In Sri Lanka, the existence of sibling species A and B was first reported by Abhayawardana et al. [7], based on the single inversion (X+^a^/X^a^) on the X chromosome. No other cytotaxonomic studies have been carried out yet to identify all the members of the *An. subpictus* complex in Sri Lanka. However, all four sibling species (A–D) have been reported to occur in Sri Lanka [8,9] based on identification using the morphometric characteristics described by Suguna et al. [6]. The method of Suguna et al. [6] was confirmed by Singh et al. [10], except for the egg morphology, and it was further concluded that a single identification character in a randomly picked individual from a population of any life stage could be used for identification of the *An. subpictus* sibling species status.

Although *An. subpictus* was described and designated as A–D, intraspecific variations within the taxon were first reported in India, among urban, pre-urban, and rural populations [11]. Kirti and Kaur [12] observed morphological differences in wings and palpi of *An. subpictus* in India. Further, a different set of morphological variations (not reported in Suguna et al. [6]), mainly in proboscis and palpi of *An. subpictus*, was reported in Sri Lanka [13,14,15]. Furthermore, the *An. subpictus* complex has been designated as only A and B in Sri Lanka based on ITS2 and cytochrome *c* oxidase I (*COI*) sequence polymorphism [16], showing the complexity of sibling species identification of the *An. subpictus* population in Sri Lanka.

Therefore, the current study was carried out to investigate the suitability of morphological character variations and genomic variations for the identification of the *An. subpictus* population in Sri Lanka.

## 2. Materials and Methods

### 2.1. Mosquito Samples

**Figure 1:**
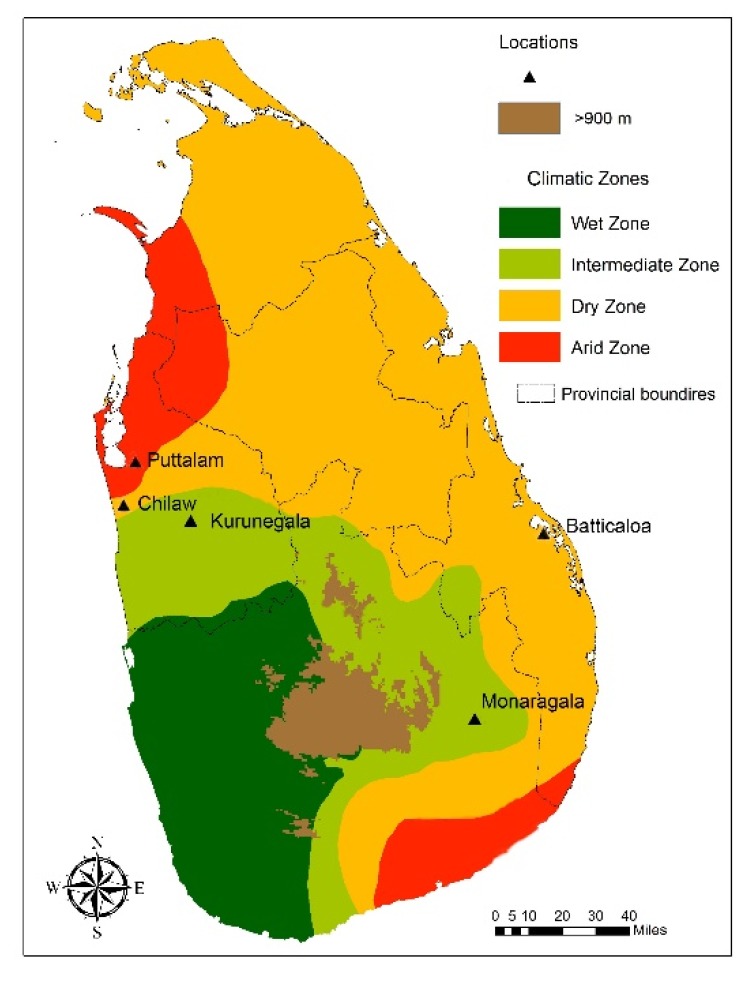
A map showing sampling sites.

Mosquito sampling sites were selected from dry and intermediate climatic zones of Sri Lanka to represent the entire *An. subpictus* population (Figure 1). Wild engorged female *An. subpictus* mosquitoes were collected from five selected different locations; Monaragala (6°87″27.02′ N, 81°35″06.70′ E), Kurunegala (7°48″71.00′ N, 80°36″49.95′ E), Puttalam (8°03″00.57′ N, 79°83″11.14′ E), Chilaw (7°57″65.41′ N, 79°79″56.57′ E), and Batticaloa (7°72″04.50′ N, 81°70″10.86′ E). Multiple collections from each location were carried out during 2012–2015. The collected mosquitoes were identified as *An. subpictus s.l.* species using standard morphological identification keys [17]. Mosquito larvae and pupae were collected from breeding sites and identified as *An. subpictus* in the laboratory using standard keys.

### 2.2. Morphological Characterization of An. subpictus Life Cycle Stages

The eggs laid by the collected adult mosquitoes were separately transferred to rearing basins and reared to obtain F_1_ progeny. Life cycle stages of eggs, fourth instar larvae, pupal exuviae, and F_1_ adults were examined from 10 randomly selected individuals in each rearing basin for the characteristic features to designate the specimen as sibling species A, B, C, or D. The observed morphological characters were mesothoracic seta 4 of larvae, seta 7-I of pupal exuviae, and proportions of apical pale and pre-apical dark bands in palpi of adults. The randomly collected larvae, pupae, and adults from the wild were examined for the morphological variations specified by Suguna et al. [6]. Individual eggs were separated into rearing basins and examined for the respective features in larvae, pupae, and adult stages. 

### 2.3. DNA Extraction, PCR Amplifications, and DNA Sequencing

Genomic DNA was extracted from F_1_ individual mosquitoes (*n* = 29) representing all adult morphological variations, using a phenol–chloroform extraction method [18]. The ITS2 region [19], D3 region [20], *white* gene [21], mitochondrial *COI* [22], and *Cyt-b* [23] were amplified in a 25 µL reaction mixture. The reaction and cyclic conditions for each PCR are shown in Table 1. Amplified PCR products were purified using QIAquick PCR product purification kit (Qiagen, Hilden, Germany) and sequenced bi-directionally at Macrogen Inc., Seoul, South Korea.

### 2.4. DNA Sequence Analysis

The sequences were assembled using the program DNA Baser v3.5.3 and were blasted over the NCBI GenBank to confirm the mosquito origin of the sequences. Open reading frames were found for the *white* gene and mitochondrial gene coding sequences and confirmed for the absence of stop codons. Sequences of each genomic region were aligned using ClustalW multiple alignment to observe the sequence polymorphism. Haplotype frequency was analyzed using DNASP 5.10.01 software. All the sequences were deposited in the NCBI GenBank.

## 3. Results

The wild-caught adult mosquitoes consisted of all sibling species A, B, C, and D in the studied population in Sri Lanka, based on the palpi polymorphism. Additionally, 66 individuals (23.0%) of the studied population showed aberrant palpi formations which could not be categorized as belonging to any of the sibling species. Percentages of each sibling species and aberrant types are shown in Table 2.

### 3.1. Sibling Species Status of Randomly Selected Life Cycle Stages; Larvae, Pupal Exuviae, and Adults 

#### 3.1.1. Larvae

**Figure 2:**
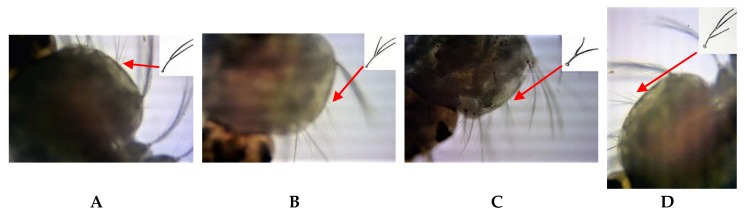
Mesothoracic seta 4 of sibling species **A**, **B**, **C**, and **D** (×200×2). Position of the setae is shown with an arrow. The shape of the setae is schematically shown [6] in the top-right corner of each figure.

Among all the observed larvae (*n* = 567), those with doubly branched mesothoracic seta 4 with a long stem were categorized as sibling species A (*n* = 218), while those with doubly branched mesothoracic seta 4 with a short stem were identified as sibling B (*n* = 67). The larvae which had triply branched mesothoracic seta 4 with the branching occurring at the same point and at two different points were classified as sibling C (*n* = 66) and D, respectively (*n* = 68). Larval mesothoracic seta IV of sibling A, B, C, and D are shown in Figure 2. One hundred and thirty-nine (139) individuals showed two different types of mesothoracic seta 4 in the left and right sides of the mesothorax. These variations have given rise to two different sibling species statuses for both the left and right sides of larvae. The remaining nine larvae showed absent or indistinguishable seta on one of the sides of the bodies.

#### 3.1.2. Pupal Exuviae

From a total of 542 pupal exuviae observed, in 142, seta 7-I was as simple and long as hairs 6 and 9 (sibling A; *n* = 142). It was 4–5 branched and shorter than hairs 6 and 9 in 75 of the pupal exuviae (sibling B; *n* = 75). Seta 7-I was medium-sized and doubly branched in 123 (sibling C; *n* = 123), while it appeared medium-sized and triply branched in 92 of the pupal exuviae (sibling D; *n* = 92). Sixty-six pupal exuviae showed two different setal morphologies in the left and right sides. Seta 7-I was indistinguishable or absent from one of the sides in 44 pupal exuviae. 

#### 3.1.3. Adults

**Figure 3:**
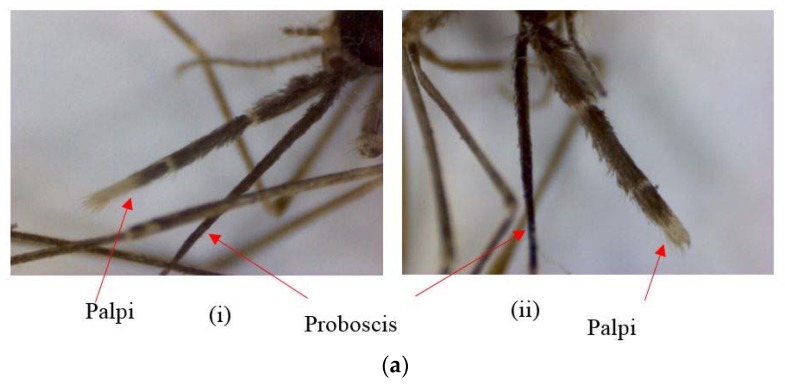

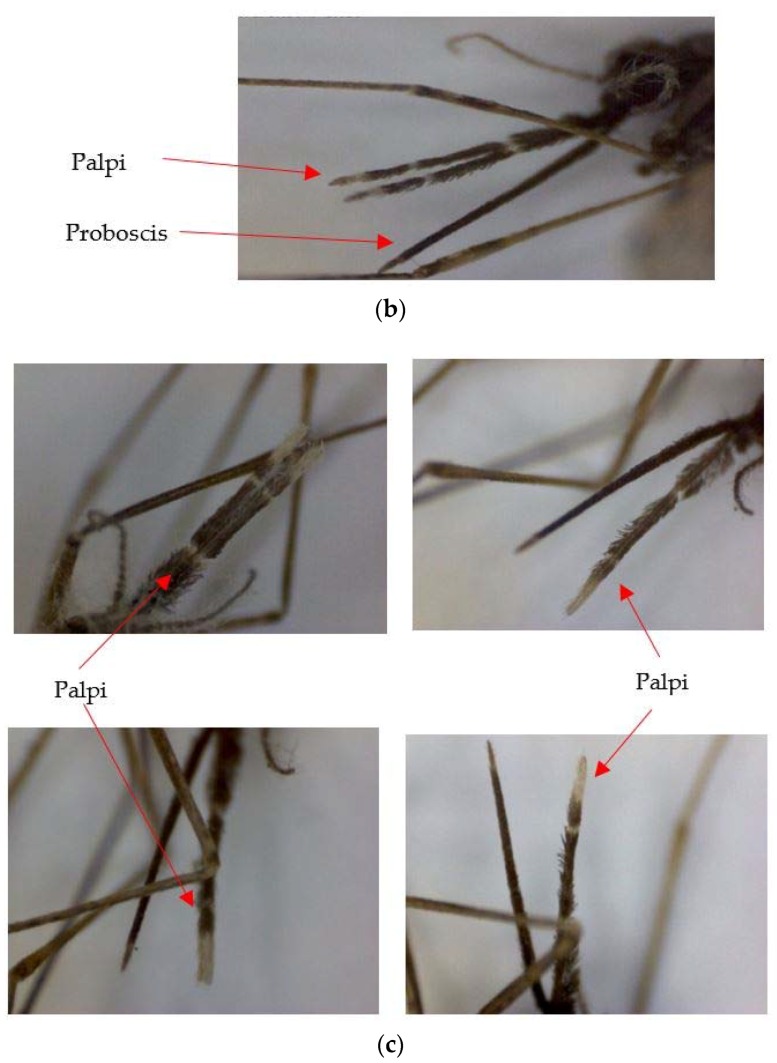
(**a**) Mosquitoes of the same isofemale progeny having (i) slightly longer preapical dark bands and (ii) slightly longer apical pale bands (×200×2); (**b**) palpi with an additional dark patch present at the tips (×200×2); (**c**) palpi with abnormally longer apical pale bands (×200×2).

Sibling species designations in adults (*n* = 523) were based on morphological differences in female palpi. Sibling A was identified for those having an apical pale band longer than the pre-apical dark band in palpi (*n* = 195), while the apical pale band was shorter than the pre-apical dark band in palpi of sibling B (*n* = 77). In sibling species C, the proboscis was longer than the palpi, with the apical pale band length equal to the pre-apical dark band length of palpi (*n* = 75). For sibling D, the proboscis and palpi were of equal length, and the apical pale band length was equal to the pre-apical dark band length in palpi (*n* = 93). Eighty-three (83) adults showed variations to the above standard features (Table 3; Figure 3). The percentage deviation from existing morphological characteristics in each life cycle stage is given in Table 4.

### 3.2. Isofemale Progeny Observation for Sibling Species Status

Among the observed 46 separate egg clutches (10 individuals per egg clutch), none of the parental females produced a single type of sibling species. Twenty individuals (43.47%) from the egg clutches produced 100% of the parental sibling status for the observed 10 individuals. However, all of these clusters showed different or a mixture of sibling characteristics in the larvae and pupal exuviae. The sibling species composition at each life stage for the observed 10 individuals from the egg clutches is shown in Table 5.

### 3.3. Examining Individual Eggs for Life Cycle Stages until Emergence

From the 98 individual eggs observed until emerging adults, only 8 individuals (10.67%) were consistent in the sibling status in consecutive larvae, pupae, and emerged adult stages. A percentage of 89.33% progeny showed different sibling characteristics in each life cycle stage. In the larvae and pupal exuviae, the occurrence of two different sibling features on the left and right sides of the body amounted to 33.33% and 26.66%, respectively (Table 6).

### 3.4. DNA Polymorphism of the An. subpictus Population in Sri Lanka

Two length variations of the ITS2 region, 480 bp and 575 bp, were identified among 29 sequences. All shorter sequences (*n* = 15) were identical, while the longer sequences (*n* = 14) were polymorphic in nine positions. All sequences were identified as having three haplotypes, in which the longer sequences encompassed two haplotypes.

All other analyzed nuclear DNA regions (D3 and *white* gene) and mitochondrial DNA regions (*COI*, *Cyt-b*) also showed two types of nucleotide sequences for all observed morphological forms. The variations were independent from the type of morphological characteristics. Further, the same sequences that shared the shorter ITS2 sequence had identical D3, *white* gene, *COI*, and *Cyt-b* sequences. Similarly, the individuals that shared the longer ITS2 sequence were in congruence with the sequence similarity in all other analyzed regions. The number of haplotypes of each genomic region and the NCBI accession numbers of the sequences are shown in Table 7.

## 4. Discussion

The results suggest that the *An. subpictus* sibling species [6] are distributed in five study locations, which are dry (Puttalam, Batticaloa) and intermediate (Chilaw, Kurunegala, Monaragala) climatic zones in Sri Lanka. The initial screening of wild-caught adult mosquitoes from the five selected locations for their morphological characteristics to determine sibling species status shows that the distribution of morphological variations is independent of the study locations. Morphological variations previously designated in the *An. subpictus* sibling species [13,14,15] and additional variations observed in the current study were commonly found from all locations. Hence, further analysis was carried out by treating the samples collected from all locations as a single pooled *An. subpictus* population from Sri Lanka.

In the morphological examination of individuals, the egg morphology was excluded due to the time restriction until hatching. The eggs were hatching on the microscopic slide, prior to completing observation of the whole egg clutch. Further, it has been shown that the eggs do not serve as a good taxonomic feature due to phenotypic plasticity [24]. Therefore, only larvae, pupal exuviae, and adult morphology were considered for further analysis.

The current study shows that morphological variations of life cycle stages previously used for sibling species characterization are unreliable and polymorphic for the population found in Sri Lanka. The percentage of variation reported from the morphological identification keys of Suguna et al. [6] was 15.87–26.10% for all life cycle stages. The extended pre-apical dark bands and the additional dark spots at the tips of palpi were first-time reports of *An. subpictus* variations, which have not been reported in India or in Sri Lanka. Furthermore, deviations of the features on either the left or right side of the larvae and pupal exuviae were observed in the present study. However, no such one-sided variations were seen in the palpus of adults; instead, all variations were indicated in the pair of palpi. Parental sibling species characteristics were not consistent for the respective F_1_ adults. Further, F_1_ adults from a single egg clutch gave rise to a mixture of sibling species, as well as individuals with features deviating from A–D sibling species. Therefore, these findings justify the unreliability of the *An. subpictus* sibling species discrimination characters [6] for the identification of the *An. subpictus* population found in Sri Lanka. Moreover, this study does not agree with the findings of Singh et al. (2010) [10], who stated the possibility of discriminating a species by a single identification character in randomly picked individuals in a population of any life stage.

In the genomic region analysis, using nuclear and mitochondrial DNA regions, two types of sequences were reported for all analyzed DNA regions. A remarkable length variation (90 bp) was observed in the ITS2 region after annotation of the sequence using the ITS2 database. The occurrence of two types of sequences was independent from the morphological variations at any life cycle stage. This finding further confirms the unsuitability of existing morphological characterization methods [6] to discriminate the *An. subpictus* species complex found in Sri Lanka. Further, the haplotype analysis showed that the shorter and longer sequence forms of the ITS2 region are two distinct sequences which were similarly separated in all studied genomic regions.

Although India is closely related and shares a biodiversity in Western Ghats that is similar to Sri Lanka, there may have been independent and distinct evolutionary forces after the main land separation during the marine introgression in the Pleistocene period. Therefore, despite the close proximity of Sri Lanka and India, the evolutionary roots and relationships of Sri Lanka to other surrounding land masses—Madagascar, Africa, Australia, and the islands of Andaman—have to be considered. In the cases of the major malaria vector of Sri Lanka, the *An. culicifacies* species complex [25], and the major vector of leishmaniasis, the *Ph. argentipus* species complex [26,27], the initial reports on the similarity of their composition to Indian species were later shown to have variations and deviations. Therefore, it is essential to consider the taxonomy and evolutionary studies for such species found in Sri Lanka without considering India as a basis. Unbiased analysis of species in Sri Lanka may reveal more biologically and evolutionarily important information on species. Further, the evolutionary patterns exerted on the two countries may have variations based on the different geographic and environmental influences. Since the geography and topology of the two land masses are different, it can be concluded that the two countries have different independent evolutionary processes for these mosquito species.

Finally, although the variations in vectorial capacity have been reported for the sibling species of the major vector species *An. culicifacies*, such variations could not be observed among the sibling species of the secondary or minor vector species of malaria. Therefore, sibling species status and the relationship of morphological variations to sibling status of *An. subpictus* should be reassessed to delineate the composition and the diversity of the *An. subpictus* species complex found in Sri Lanka.

## 5. Conclusions

The *An. subpictus* population in Sri Lanka could not be identified as a sibling species using their previously reported morphological characteristics. In addition, the genomic variations found in the study are independent from the morphological variations of the *An. subpictus* population found in Sri Lanka.

## Figures and Tables

**Table 1:** Reactions and cyclic conditions for the PCR amplifications.

Genomic Region	Reaction Conditions	Cyclic Conditions	
ITS2	1X PCR buffer	5 min at 94 °C	
	1.25U of *Taq* DNA Polymerase (Promega)	1 min at 95 °C	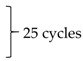
	1.5 mM MgCl_2_, 0.2 mM dNTP	2 min at 50 °C	
	50 ng of primers	2 min at 72 °C	
	10 ng of template DNA	7 min at 72 °C	
D3	1X PCR buffer	5 min at 94 °C	
	1.25U of *Taq* DNA Polymerase (Promega)	30 s at 95 °C	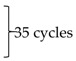
	1.5 mM MgCl_2_, 0.2 mM dNTP	30 s at 50 °C	
	50 ng of primers	45 s at 72 °C	
	10 ng of template DNA	7 min at 72 °C	
*white* gene	1X PCR buffer	5 min at 94 °C	
	1.25U of *Taq* DNA Polymerase (Promega)	1 min at 95 °C	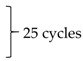
	1.5 mM MgCl_2_, 0.2 mM dNTP	2 min at 50 °C	
	25 pmol of primers	2 min at 72 °C	
	10 ng of template DNA	7 min at 72 °C	
cytochrome *c* oxidase I (*COI*) and *Cyt-b*	1X PCR buffer	5 min at 94 °C	
	1.20U of *Taq* DNA Polymerase (Promega)	30 s at 94 °C	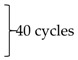
	1.5 mM MgCl_2_, 0.2 mM dNTP	30 s at 45 °C	
	50 ng of primers	40 s at 72 °C	
	10 ng of template DNA	10 min at 72 °C	

**Table 2:** Sibling species status of wild-caught female mosquitoes from sampling sites.

Location	Total Number of Mosquitoes	Sibling Status of Wild-Caught Female Mosquitoes Based on Suguna et al. [6]								Unclear or Not Recognized	
		A		B		C		D			
Puttalam	64	18	28.1%	12	18.8%	12	18.8%	11	17.2%	11	17.2%
Chilaw	29	11	37.9%	5	17.2%	3	10.3%	2	6.9%	8	27.6%
Batticaloa	51	15	29.4%	10	19.6%	6	11.8%	2	3.9%	18	35.3%
Monaragala	75	13	17.3%	27	36.0%	11	14.7%	11	14.7%	13	17.3%
Kurunegala	68	25	36.8%	5	7.3%	4	5.9%	18	26.5%	16	23.5%
Total	287	82	28.6%	59	20.6%	36	12.5%	44	15.3%	66	23.0%

**Table 3:** Additional palp variations of adults.

Feature	Slightly Longer Pre-Apical Dark Bands	Slightly Longer Apical Pale Bands	Additional Dark Patch on the Tip of Palpi	Abnormally Longer Apical Pale Bands (2 Times of the Peripheral Dark Band)
Number of individuals	14	20	09	40

**Table 4:** Percentages of deviations from existing morphological discrimination characteristics (Suguna et al. [6]).

Life Cycle Stage	Number of Deviation/Total Observed	Percentage
Larvae	148/567	26.10%
Pupal exuviae	110/542	20.29%
Adult	83/523	15.87%

**Table 5:** Morphological identification of sibling species status of parental female, larvae, pupal exuviae, and F_1_ adults.

Isofemale Progeny No.	Sibling Species Status of Parental Female	Sibling Species Status of Larvae in the Progeny (Observed Number)	Sibling Species Status of Pupae in the Progeny (Observed Number)	Sibling Species Status of F_1_ Adults (Observed Number)
1	A	A(10)	B(10)	A(10)
2	A	A(10)	C(10)	A(10)
3	B	A(6), D(3), A/D(1)	D(4), A/C(3), D/C(3)	D(10)
4	A	A(7), D(3)	A(6), C(4)	A(10)
5	A	C(10)	C(10)	A(10)
6	A	C/D(6), D(4)	C(5), C/D(5)	A(10)
7	A	A(5), A/D(5)	B(10)	A(6), B(4)
8	A	A(10)	B(6), UN(4)	B(10)
9	A	B(8), A/D(2)	D(3), C(3), A/B(2), A/D(2)	A(6), C(4)
10	C	A/D(6), D(4)	C(3), D(4), A/D(3)	C (5), D(5)
11	C	A(10)	D(10)	A(10)
12	A	A/D(10)	A(6), UN(4)	A(6), UN(4)
13	B	B(10)	A(5), B(3), C/D(2)	C(7), D(3)
14	B	A/D(10)	A(7), C(3)	A*(10)
15	B	A(3), D(7)	C(6), D(4)	C(10)
16	B	A(10)	A(5), B(4), NR(1)	A(10)
17	A	A/C(10)	A(6), C(4)	D(10)
18	C	A(10)	A(5), UN(5)	A(10)
19	B	B(8), C(2)	A(10)	B(10)
20	C	D(6), A/C(1), C(3)	C(10)	A(8), C(2)
21	C	C(10)	A(7), UN(3)	A(8), D(2)
22	C	C/D(10)	A(8), C/D(2)	C(10)
23	B	C(10)	C(5), D(5)	B(10)
24	B	A/D(10)	A(4), B(4), UN(2)	A(6), C(4)
25	NR	A(10)	A(4), B(4), D(2)	A(6), D(2)
26	B	A/D(10)	C(10)	B(10)
27	A	A(10)	B(10)	D(10)
28	A	B(10)	C(5), D(4), C/D(1)	A(10)
29	A	A(10)	C(7), D(3)	A(10)
30	C	C(10)	B(10)	D(10)
31	C	B(10)	C(10)	C(10)
32	A	A(10)	A(7), UN(3)	A(10)
33	B	A/D(10)	A(4), B(3), UN(3)	B*(10)
34	D	A(4), D(3), A/D(3)	A(8), A/C(2)	A(5), B(4), C(1)
35	C	A(10)	C(10)	C(10)
36	B	A(10)	A(7), A/C(3)	C(10)
37	A	A(10)	A(7), UN(3)	A(10)
38	B	A/B(8), B/D(2)	D(6), B/D(4)	A*(10)
39	A	A(10)	A(7), UN(3)	A(10)
40	B	B(10)	D(10)	B(10)
41	D	D(6), C/D(4)	D(6), C/A(3), C/D(1)	B(10)
42	C	C(10)	C(6), D(4)	D*(10)
43	A	C(7), A/C(3)	A(6), UN(4)	A(10)
44	D	D(10)	D(6), C/D(4)	C(10)
45	A	A(4), D(3), A/D(2), C/D(1)	C(5), B/D(5)	A(10)
46	D	D(10)	A(3), A/D(3), C/D(2), B(2)	D(10)

Sibling species status due to occurrence of different features at both sides of larvae and pupae are separated by a slash (/), while the different sibling species status encountered in different individuals are separated by a comma (,). The numbers in parenthesis show the number of individuals that were identified by the indicated sibling species status. Deviated characteristics are shown as UN. Asterisk (*) indicates exceptional dark scales on the wing.

**Table 6:** Morphological identification of sibling species status of larvae, pupae, and F_1_ adults of individual eggs in five isofemale progenies.

Individual No.	Larval Sibling Species Designation	Pupal Sibling Species Designation	F_1_ Adult Sibling Species Designation
1	UN	D	A
2	A	A	A
3	A	A/D	D
4	A	A/B	B
5	A/B	A	A
6	A	C	A
7	C	D	A
8	C	C	D
9	A	A/C	C
10	C	A/C	D
11	A	D	D
12	A	A	D
13	A	D	A
14	A	B	D*
15	B	UN	D
16	D/UN	UN	D
17	A/UN	A	D
18	B	C	C
19	A/B	D	A
20	A/UN	A	D
21	C/D	D	D
22	A	D	A
23	A	A	D
24	A	B	B
25	A	D	A
26	A/C	A	A
27	A	B	A
28	A	B	A
29	A	A	A*
30	A	A	A
31	A	A	D*
32	A	A	D
33	A/D	A/C	A
34	B/D	C	B
35	A/D	A/C	A
36	A/D	D	A
37	A	B	A
38	D	D	D
39	B/D	D	A
40	A/D	D	D
41	A/UN	A	A
42	D	A/C	A
43	A	D/absent	UN
44	D	C	A
45	D	A/C	UN
46	C/D	A/C	A
47	A	A/C	UN
48	A	A	A
49	A	B/C	A
50	A	A	A
51	A/UN	D	A
52	C/D	A/C	A
53	A/C	C	A
54	B	B	B
55	B	C/absent	UN
56	A	D	A*
57	B	D	D
58	A/B	A/C	A
59	A/D	A	A
60	A	A	D*
61	A	D	D*
62	A	B	B
63	A/D	D	D
64	A	A	A*
65	A/B	D	A
66	A	A	A
67	A	A/B	A*
68	A	D	D
69	A/D	B/C	A*
70	D	A	A
71	B/C	A/C	A*
72	A/B	A/C	A
73	B	C	A*
74	B	C/absent	A*
75	B	B	A*

Different features on both sides of larvae and pupae are separated by a slash (/) and the deviated characteristics are shown as UN. Asterisk (*) indicates exceptional dark scales on the wing.

**Table 7:** Number of haplotypes of each genomic region and the accession numbers received from the NCBI GenBank.

Genomic Region	No. of Haplotypes	NCBI GenBank Accession Numbers
Nuclear		
ITS2	3	KP165072–KP165079
D3	2	KT285501–KT285508
*White* gene	2	KP733780–KP733793
Mitochondrial		
*COI*	11	KJ461779–KJ461791
*Cyt-b*	10	KT285491–KT285500

## References

[1] Amerasinghe P.H., Amerasinghe F.P., Wirtz R.A., Indrajith N.G., Somapala W., Pereira L.R., Rathnayake A.M.S. (1992). Malaria transmission by *Anopheles subpictus* (Diptera: Culicidae) in a New Irrigation Project in Sri Lanka. J. Med. Entomol..

[2] Chandra G., Bhattacharjee I., Chatterjee S. (2010). A review on *Anopheles subpictus* Grassi-A biological vector. Acta Trop..

[3] Wickramasinghe M.B. (1981). Malaria and its control in Sri Lanka. Ceylon Med. J..

[4] Reid J.A. (1966). A Note on *Anopheles subpictus* Grassi and *Anopheles indefinitus* Ludlow (Diptera: Culicidae). J. Med. Entomol..

[5] Reuben R., Suguna S.G. (1983). Morphological differences between sibling species of the taxon *Anopheles subpictus* Grassi in India, with notes on relationships with known forms. Mosq. Syst..

[6] Suguna S.G., Rathinam K.G., Rajavel A.R., Dhanda V. (1994). Morphological and chromosomal descriptions of new species in the *Anopheles subpictus* complex. Med. Vet. Entomol..

[7] Abhayawardana T.A., Wijesuriya S.R.E., Dilrukshi R.K.C. (1996). *Anopheles subpictus* Complex: Distribution of Sibling species in Sri Lanka. Indian J. Malariol..

[8] Abhayawardana T.A., Wickramasinghe M.B., Amerasinghe F.P. (1999). Sibling species of *Anopheles subpictus* and seasonal abundance in Chilaw area. Proc. Sri Lanka Assoc. Adv. Sci..

[9] Surendran S.N., Ramasamy R. (2010). The *Anopheles culicifacies* and *Anopheles subpictus* species complexes in Sri Lanka and their implications for malaria control in the island. Trop. Med. Health.

[10] Singh S.P. (2014). Morphotaxonomical studies to identify the member of the *Anopheles subpictus* Grassi (Diptera: Culicidae) species complex in villages of District Mewat Haryana State, India. Online Int. Interdiscip. Res. J..

[11] Banerjee P.K., Chatterjee R.N. (1997). A study of biology of *A. subpictus* in West Bengal with reference of its intraspecific variation between urban and peri-urban and rural population. Proc. Zool. Soc. India.

[12] Kirti J.S., Kaur J. (2004). Variations in ornamentation of wings and palpi of *Anopheles* (*Cellia*) *subpictus* Grassi collected from northwest India. J. Vector Borne Dis..

[13] Gunathilaka P.A.D.H.N., Fernando M.A.S.T., Premasiri D.S., Hapugoda M.D., Wijeyerathne P., Wickremasinghe A.R., Abeyewickreme W. Morphological differences among *Anopheles subpictus* sibling species B breeding in waste water habitats in Mannar District, Sri Lanka. Proceedings of the 12th Annual Research Symposium.

[14] Jude P.J., Ramasamy R., Surendran S.N. (2014). Bionomic aspects of the *Anopheles subpictus* species complex in Sri Lanka. J. Insect Sci..

[15] Chhilar J.S., Chaudhry S. (2012). Phylogenetic analysis of *Anopheles* (*Cellia*) *subpictus* Grassi using rDNA-ITS2 sequence. Proc. Zool. Soc..

[16] Surendan S.N., Sarma D.K., Jude P.J., Kemppainen P., Kanthakumaran N., Gajapathy K., Peiris L.B.S., Ramasamy R., Walton C. (2013). Molecular characterization and identification of members of the *Anopheles subpictus* complex in Sri Lanka. Malar. J..

[17] Amerasinghe F.P. (1990). A Guide to the Identification of the Anopheline Mosquitoes (Diptera: Culicidae) of Sri Lanka, I. Adult Females. Ceylon J. Sci. Biol. Sci..

[18] Ballinger-Crabtree M.E., Black W.C., Miller B.R. (1992). Use of genetic polymorphisms detected by the Random-Amplified Polymorphic DNA Polymersae Chain Reaction (RAPD PCR) for differentiation and identification of *Aedes aegypti* subspecies and populations. Am. J. Trop. Med. Hyg..

[19] Dezfouli S.R.N., Oshaghi M.A., Vatandoost H., Assmar M. (2003). rDNA-ITS2 based species-diagnostic Polymerase Chain Reaction assay for identification of sibling species of *Anopheles fluviatilis* in Iran. Southeast Asian J. Trop. Med. Public Health.

[20] Jariyapan N., Choochote W., Junkum A., Jitpakdi A., Komalamisra N., Bates P.A., Crampton J.M. (2005). Sequence analyses of three nuclear ribosomal loci and a mitochondrial locus in cytologically different forms of Thai *Anopheles aconitus* mosquitoes. Southeast Asian J. Trop. Med. Public Health.

[21] Reidenbach K.R., Cook S., Bertone M.A., Harbach R.E., Wiegmann B.M., Besansky N.J. (2009). Phylogenetic analysis and temporal diversification of mosquitoes (Diptera: Culicidae) based on nuclear genes and morphology. BMC Evol. Biol..

[22] Simon C., Frati F., Beckenbach A., Crespi B., Liu H., Flook P. (1994). Evolution, weighting, and phylogenetic utility of mitochondrial gene sequences and a compilation of conserved polymerase chain reaction primers. Ann. Entomol. Soc. Am..

[23] Dusfour I., Linton Y.M., Cohuet A., Harbach R.E., Baimai V., Trung H.D., Chang M.S., Matusop A., Manguin S. (2004). Molecular evidence of speciation between island and continental populations of *Anopheles* (*Cellia*) *sundaicus* Rodenwaldt (Diptera: Culicidae), a principal malaria vector in Southeast Asia. J. Med. Entomol..

[24] Gakhar S.K., Sharma R., Sharma A. (2013). Population genetic structure of malaria vector *Anopheles stephensi* Liston (Diptera: Culicidae). Indian J. Exp. Biol..

[25] Surendran S.N., Truelove N., Sarma D.K., Jude P.J., Ramasamy R. (2015). Karyotypic assignment of Sri Lankan *Anopheles culicifacies* species B and E does not correlate with cytochrome oxidase subunit I and microsatellite genotypes. Parasite Vectors.

[26] Gajapathy K., Jude P.J., Surendran S.N. (2011). Morphometric and meristic characterization of *Phlebotomus argentipes* species complex in northern Sri Lanka: Evidence for the presence of potential leishmaniasis. Trop. Biomed..

[27] Gajapathy K., Tharmasegaram T., Eswaramohan T., Peries L.B.S.L. (2016). DNA barcoding of Sri Lankan phlebotomine sand flies using cytochrome c oxidase subunit I reveals the presence of cryptic species. Acta Trop..

